# Resistance to cisplatin and paclitaxel does not affect the sensitivity of human ovarian cancer cells to antiprogestin-induced cytotoxicity

**DOI:** 10.1186/1757-2215-7-45

**Published:** 2014-04-27

**Authors:** Carlos D Gamarra-Luques, Maria B Hapon, Alicia A Goyeneche, Carlos M Telleria

**Affiliations:** 1 Division of Basic Biomedical Sciences, Sanford School of Medicine, The University of South, Dakota, 414 East Clark Street, Vermillion, SD 57069, USA; 2 Present Address: Institute of Medicine and Experimental Biology of Cuyo, National Council for Scientific and Technical Research (CONICET), Mendoza, Argentina

**Keywords:** Ovarian cancer, Chemoresistance, Antiprogestins

## Abstract

**Background:**

Antiprogestin compounds have been shown to be effective in blocking the growth of ovarian cancer cells of different genetic backgrounds. Herein we studied the anti-ovarian cancer effect of a series of antiprogestins sharing the chemical backbone of the most characterized antiprogestin, mifepristone, but with unique modifications in position C-17 of the steroid ring. We assessed the effect of mifepristone-like antiprogestins on the growth of ovarian cancer cells sensitive to the standard combination therapy cisplatin-paclitaxel or made double-resistant upon six cycles of pulse-selection with the drugs used at clinically relevant concentrations and exposure times.

**Methods:**

IGROV-1 and SKOV-3 cells were pulsed with 20 μM cisplatin for 1 h followed by 100 nM paclitaxel for 3 h once a week for six weeks. The cells that did not die and repopulate the culture after the chemotherapies were termed Platinum-Taxane-EScape cells (PTES). Parental cells were compared against their PTES derivatives in their responses to further platinum-taxane treatments. Moreover, both ovarian cancer cells and their PTES siblings were exposed to escalating doses of the various antiprogestin derivatives. We assessed cell growth, viability and sub-G1 DNA content using microcapillary cytometry. Cyclin-dependent kinase inhibitors p21^cip1^ and p27^kip1^ and cleavage of downstream caspase-3 substrate PARP were used to assess whether cell fate, as a consequence of treatment, was limited to cytostasis or progressed to lethality.

**Results:**

Cells subjected to six pulse-selection cycles of cisplatin-paclitaxel gave rise to sibling derivatives that displayed ~2-7 fold reduction in their sensitivities to further chemotherapy. However, regardless of the sensitivity the cells developed to the combination cisplatin-paclitaxel, they displayed similar sensitivity to the antiprogestins, which blocked their growth in a dose-related manner, with lower concentrations causing cytostasis, and higher concentrations causing lethality.

**Conclusions:**

Antiprogestins carrying a backbone similar to mifepristone are cytotoxic to ovarian cancer cells in a manner that does not depend on the sensitivity the cells have to the standard ovarian cancer chemotherapeutics, cisplatin and paclitaxel. Thus, antiprogestin therapy could be used to treat ovarian cancer cells showing resistance to both platinum and taxanes.

## Background

Epithelial ovarian carcinoma is a highly lethal disease, mostly a consequence of its frequent detection at an advanced stage and its ability to metastasize within the peritoneal cavity. Debulking surgery followed by platinum-taxane based chemotherapy is the standard of care for patients with advanced stage ovarian cancer. However, despite an encouraging response rate of 65%–80% to first-line chemotherapy, most patients relapse with chemoresistant disease. This presents a challenge in the clinic as no reliable second-line therapies have been shown to be a suitable success for these patients, leading to a lack of remarkable improvements in the cure rate over the past thirty years (rev.in [1-6]).

Following surgery and platinum-taxane treatment patients are not usually further treated until recurrence is clinically evident. Thus, one strategy worth studying is the development of chronic therapeutic approaches to follow standard front-line therapy for ovarian cancer patients. One such possibility for maintenance therapy is the use of antiprogestin compounds that can be chronically given with minimal toxicity [7]. Our laboratory studied the effect of antiprogestin mifepristone, which successfully blocked the growth of ovarian cancer cells in vitro and in vivo [8], and prevented the repopulation of ovarian cancer cells that escaped cisplatin (CDDP) [9] or CDDP-paclitaxel (PTX) [10] therapies. We have also found that antiprogestins mifepristone, ORG-31710, and ulipristal (CDB-2914), when used at pharmacologic concentrations, cause cytostasis by blocking the activity of cyclin-dependent kinase 2 (Cdk2), thus preventing the cells from moving towards the G1/S transition, and, consequently, synthesizing DNA; moreover, these compounds increased the accumulation of cyclin-dependent kinase inhibitors p21^cip1^ and p27^kip1^, as well as promoted their association to Cdk2, leading to its reduced activity [8,11].

**Figure 1:**
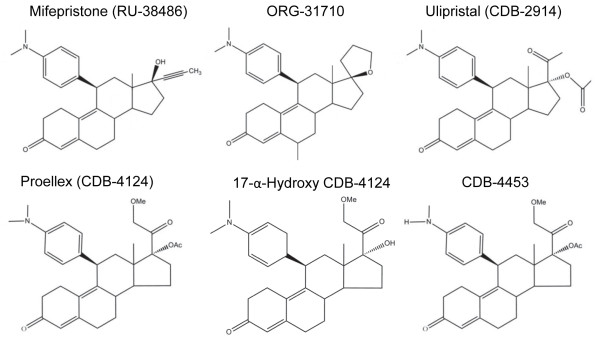
Chemical structure of antiprogestins used in the study.

If antiprogestin therapy to control ovarian cancer repopulation or recurrence following initial standard platinum-taxane chemotherapy is to be used, it would almost always encounter cells that have escaped chemotherapy and consequently acquired various degrees of resistance to the front-line platinum and taxane derivatives. Herein we set up to study whether mifepristone and a group of mifepristone-related compounds with unique modifications in position C-17 of the steroid ring (portrayed in Figure 1) are capable of abrogating the growth of ovarian cancer cells that developed clinically relevant resistance to CDDP and PTX.

## Methods

### Cell culture and treatments

The human ovarian carcinoma cell lines SKOV-3 and IGROV-1 were obtained from the American Type Culture Collection (ATCC, Manassas, VA) and the laboratory of Dr. Howell (University of California, San Diego), respectively. Cultures were propagated under conditions previously described in detail [10,11].

Cisplatin (CDDP; cis-diamminedichloroplatinum II) (Sigma Chemical Co, St Louis, MO) was prepared fresh in 0.9% NaCl every time it was used. A stock of 100 μM paclitaxel (PTX; Sigma) was prepared in DMSO and was stored at -20°C.

Mifepristone was commercially obtained (Sigma). ORG-31710 was provided by N.V. Organon (Oss, The Netherlands). Ulipristal (a.k.a. CDB-2914) was provided by HRA Pharma (Paris, France). Proellex (a.k.a. CDB-4124), 17α-hydroxy CDB-4124 (17α-hydroxy-proellex), and CDB-4453 (mono-demethylated CDB-4124) were kindly provided by Repros Therapeutics, Inc (The Woodlands, TX). The antiprogestins were prepared as a stock 20 mM solution in DMSO and stored at -20°C. The maximum concentration of DMSO reached in the culture was 0.2% (v/v).

### Cell proliferation and viability

Following the indicated treatments, triplicate cultures were trypsinized, pelleted by centrifugation at 500 *g* for 5 min, and washed with PBS. The cells were resuspended in ViaCount reagent (Guava Technologies, Hayward, CA) and studied using the Guava ViaCount application in the Guava EasyCyte Mini microcapillary cytometer (Guava Technologies) as we previously reported [9]. When indicated, the concentration of drugs that caused inhibition of 50% in growth (IC50) were determined using software designed to study drug interaction, which calculates the median effective dose or Dm that is similar to the IC50 (Calcusyn, Biosoft, Cambridge, UK).

### Generation of platinum–taxane escape (PTES) cells

**Figure 2:**
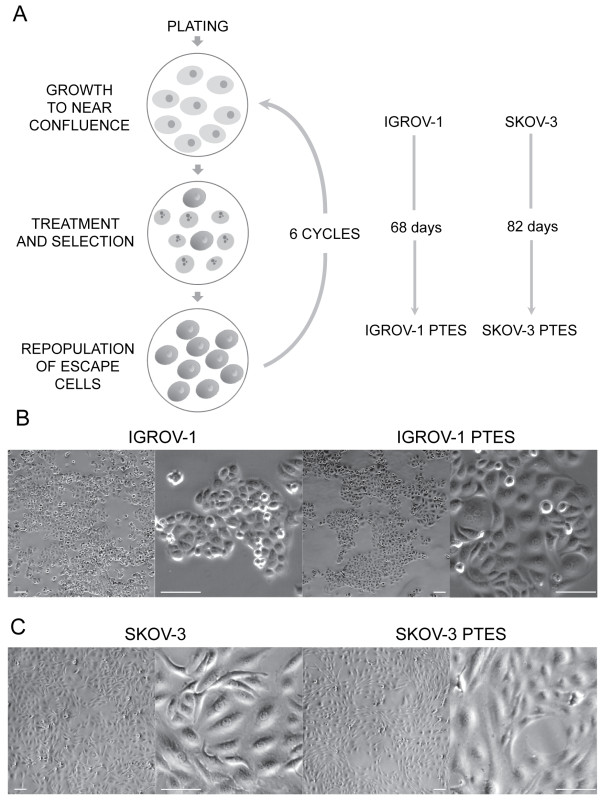
**Generation of ovarian cancer cells resistant to CDDP and PTX. (A)** Graphical representation of the procedure performed to generate cells with lower sensitivity to both CDDP and PTX. Lighter cells represent growing cells whereas darker cells are cells that survive therapy. Cells showing nuclear fragmentation represent those dying in response to chemotherapy. Phase contrast images at lower or higher magnifications of the morphologies displayed by IGROV-1 and the IGROV-1 PTES **(B)** and that of SKOV-3 and SKOV-3 PTES siblings **(C)**. Scale bar, 100 μm.

Ovarian carcinoma IGROV-1 and SKOV-3 cells were plated into T75 cm^2^ culture flasks. When the culture reached 90% confluence, the cells received one chemotherapeutic challenge consisting of 20 μM CDDP for 1 h followed by 100 nM PTX for 3 h, which was repeated weekly for six weeks. Upon the repopulation following the last chemotherapeutic challenge, the cells were considered as Platinum-Taxane-EScape cells (PTES), and were trypsinized and stored in liquid nitrogen for subsequent uses. Figure 2A displays a schematic summary of the experimental procedure implemented.

### Determination of sub-G1 DNA content

After 96 h of the indicated treatments, the cells were trypsinized, pelleted, washed, fixed and analyzed by microcytometry as we previously described in detail [10].

### Western blotting

After 48 h of the indicated treatments, the cells were harvested, washed with PBS, pelleted and maintained at -80°C until further use. The preparation of the cell lysates for gel electrophoresis has been detailed previously [12]. Primary antibodies for the following proteins were used at the indicated dilutions: p21^cip1^ (clone 6B6; 2 μg/ml) and cyclin E (clone HE12; 0.5 μg/ml), were from BD Pharmigen (San Diego, CA); p27^kip1^ (clone 57; 1:2,000) was from BD Transduction Laboratories (San Diego, CA); Cdk2 (M2; 1:1,000) and HSC-70 (sc-7298; 1:5,000) were from Santa Cruz Biotechnology (Santa Cruz, CA); and poly (ADP- ribose) polymerase (PARP) (#9542; 1:1000) was from Cell Signaling Technologies (Danvers, MA).

## Results

### Generation of ovarian cancer cells with clinically relevant resistance to CDDP and PTX

We used pulse-selection with clinically relevant doses and exposure times of CDDP and PTX to develop two ovarian cancer cells lines with double resistance that would reflect the clinical setting. We selected two cell lines with different genetic backgrounds and known initial sensitivities to CDDP and PTX, and pulse-challenged them with concentrations and times of exposure of the drugs resembling those used in the clinic. To pulse the cells we chose 1 h exposure to CDDP and 3 h exposure to PTX, which are the times patients receive the chemotherapeutics in tandem intravenously [13]. Furthermore, we selected 20 μM CDDP which is the peak plasma level reached following an intravenous bolus of 100 mg/m^2^ CDDP [14]. In terms of PTX, we selected 100 nM because this concentration can be reached when the agent is given at a dose of 175 mg/m^2^[15]. This is an approximation without considering the metabolism of the drugs in the body, yet we mimic the clinic by exposing the cells to the drugs for a maximum of only 1 h for CDDP and 3 h for PTX. We also simulated the six cycles of chemotherapy received by patients by allowing one week recovery in between each pulse/challenge with the drugs. Thus, we exposed IGROV-1 and SKOV-3 cells to weekly rounds of combination therapy consisting of 20 μM CDDP for 1 h followed by 100 nM PTX for 3 h. Each cycle was followed by culture in drug-free media with the media being replaced every two days. After six cycles of treatment, the sibling cells that still survived and escaped the chemotherapy were termed, respectively, IGROV-1 PTES and SKOV-3 PTES, where PTES means Platinum-Taxane-EScape cells. These cells were considered as in vitro recurrent (Figure 2A). When compared to the parental IGROV-1 cells, the PTES siblings had lesser tendency to growth in layers, displayed larger cytoplasm, and showed frequent multi-nucleation (Figure 2B). As for the PTES derivatives of SKOV-3, they displayed extended cytoplasm and seemed flattened when compared to their parental counterparts (Figure 2C).

### Ovarian cancer cells escaping six cycles of CDDP-PTX therapy have reduced sensitivity to a further round of chemotherapy

We next confirmed whether the sibling cells that had regrown after surviving six rounds of CDDP-PTX therapy developed reduced sensitivity to an additional chemotherapeutic challenge, when compared to their parental counterparts. We tested the growth of cells after a single exposure to increasing doses of CDDP, PTX, or a combination of both. To simplify the presentation of data when CDDP and PTX were combined in different doses, we defined the concept of Combination Dose Proportion (CDP). We termed CDP a combination of doses and exposure times that when equal to 1 are within the range of clinical achievability. Thus, a CDP of 1 means 20 μM CDDP for 1 h plus 100 nM PTX for 3 h. Accordingly, for instance, a CDP of 0.5 means that the inhibition of growth by 50% was achieved using half the concentration of each of the drugs (in this case 10 μM CDDP for 1 h plus 50 nM PTX for 3 h).

**Figure 3:**
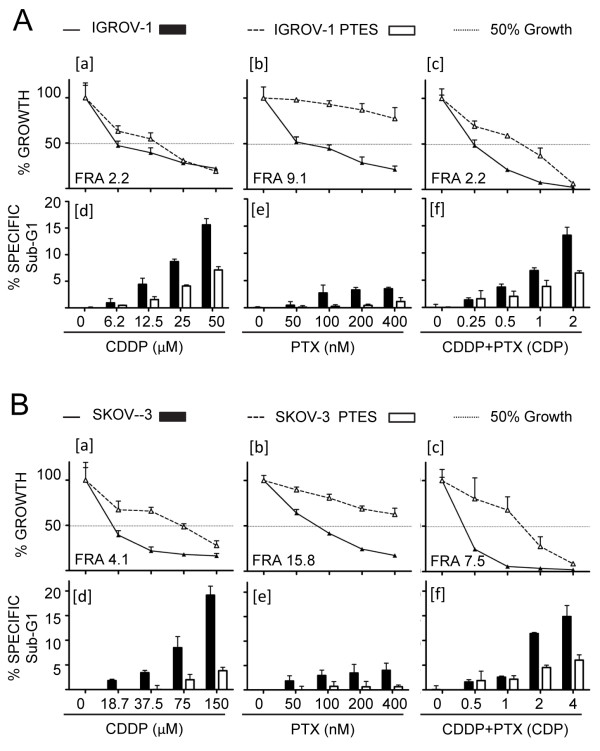
**Characterization of ovarian cancer cells made resistant to CDDP and PTX. (A)** Percent of cell growth [a-c] and sub-G1 DNA content [d-f] in IGROV-1 and IGROV-1 PTES cells. **(B)** Percent of cell growth [a-c] and sub-G1 DNA content [d-f] in SKOV-3 and SKOV-3 PTES cells. Percent growth was expressed in relation to the growth of vehicle treated controls considered as 100%. Percent specific sub-G1 was calculated by the following equation: specific sub-G1 = [100* (sub-G1 treatment – sub-G1 control) / (100 - control sub- G1)]. Data presented in panels [a-f] were collected after 96 h treatment. FRA; fold resistance acquired; CDP: combination dose proportion, where CDP 1 represents 20 μM CDDP + 100 nM PTX, CDP 0.25 represents 5 μM CDDP + 25 nM PTX, CDP 0.5 represents 10 μM CDDP + 50 nM PTX, whereas CDP 2 represents 40 μM CDDP + 200 nM PTX.

In terms of growth inhibition, IGROV-1 PTES required 2.2 fold higher concentration of CDDP and 9.1 fold higher concentration of PTX to have their replication rate diminished by 50% (IC50), which is depicted as fold resistance acquired (FRA; Figure 3A, panels [a] and [b] and Table 1). When CDDP and PTX were combined, the doses needed to block 50% of growth increased by 2.2 fold (Figure 3A, panel [c] and Table 1). The induction of cell death was assessed for the previous treatment approaches four days after drug exposure by quantifying the percentage of cellular particles with DNA content below G1, which is indicative of cells undergoing nuclear fragmentation during apoptotic death. We observed that IGROV-1 PTES required higher concentrations of CDDP, PTX, and of the combination of CDDP plus PTX, in order to reach the damage done by much lower doses of the drugs in the parental cells (Figure 3A, panels [d], [e] and [f]). Similarly to IGROV-1 PTES, when compared to the parental SKOV-3 cells, SKOV-3 PTES displayed 4.1 fold reduction in their sensitivity to CDDP (Figure 3B, panel [a]), 15.8 fold reduction in sensitivity to PTX (Figure 3B, panel [b]), and a global 7.5 fold reduction in sensitivity to the combination CDDP/PTX (Figure 3B, panel [c]). The reduced sensitivity to the chemotherapeutic agents was further corroborated in terms of reduced sub-G1 DNA content (Figure 3B, panels [d-f].

**Table 1:** Development of cells resistant to CDDP and PTX

**Cells**	**IC50 CDDP (μM)**	**IC50 PTX (nM)**	**CDP (CDDP + PTX)**
IGROV-1	5.40 ± 0.57	60.9 ± 1.22	0.24 ± 0.04
IGROV-1 PTES	12.1 ± 1.01^b^	554 ± 15.5^b^	0.52 ± 0.04^b^
SKOV-3	15.6 ± 1.20	78.0 ± 6.40	0.16 ± 0.20
SKOV-3 PTES	64.1 ± 4.10^a^	1230 ± 300^c^	1.20 ± 0.20^c^

CDP = Combination Dose Proportion. CDP is considered a combination of doses and exposure times that when equal to 1 are clinically achievable (CDP = 1 means 20 μM CDDP for 1 h plus 100 nM PTX for 3 h). For instance, a CDP of 0.5 means that the inhibition of growth by 50% was achieved using half the concentration of each of the drugs (i.e., 10 μM CDDP for 1 h plus 50 nM PTX for 3 h). ^a^p < 0.001; ^b^p < 0.01; ^c^p < 0.05 (Student’s *t*-test) compared to parental cells.

### Although with different potencies, antiprogestins block growth of ovarian cancer cells regardless of their sensitivities to the combination CDDP-PTX

We next studied the responses of IGROV-1 and SKOV-3 cells and their respective, less chemosensitive siblings IGROV-1 PTES and SKOV-3 PTES, to a panel of antiprogestin derivatives. Previously we have shown, using dose-response studies, that antiprogestin RU-38486 (mifepristone), ORG-31710, and CDB-2914 (ulipristal) have cytostatic effects at lower doses and lethal effects at higher concentrations. Herein, in addition to those three antiprogestins, we studied CDB-4124 (proellex) and two of its derivatives, 17-α-hydroxy CDB-4124 and CDB-4453, the latter carrying a de-methylation in position 11 (Figure 1). We assessed whether the panel of antiprogestins are able to display their cytotoxic effect, either cytostasis or lethality, in cells that had been made simultaneously resistant (i.e., double resistant) to CDDP and PTX. Four days after treatment with the various antiprogestins, we evaluated the responses of the cells in terms of growth in culture by measuring cell number and of lethality by assessing cell viability and sub-G1 DNA content.

**Figure 4:**
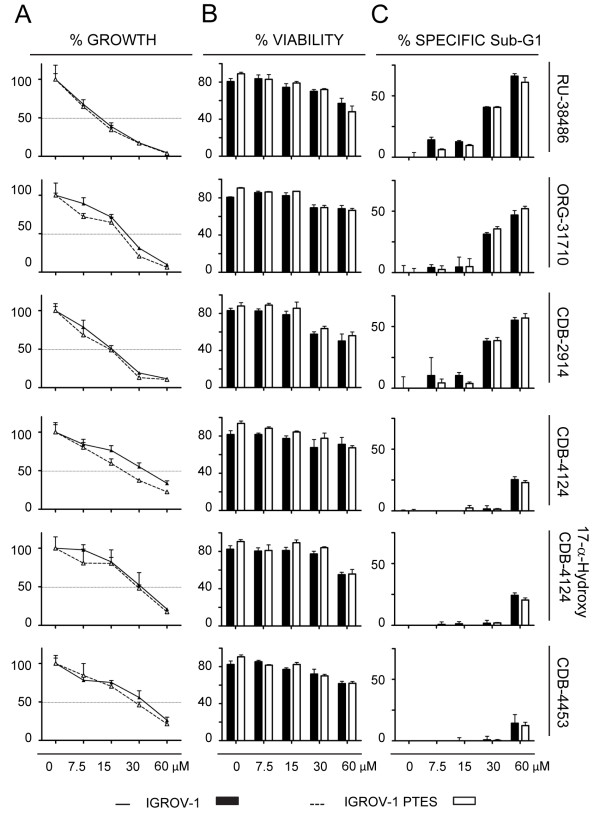
**Growth inhibition and lethality of antiprogestins towards IGROV-1 and IGROV-1 PTES cells.** Cells were plated in equal numbers, allowed to attach to the surface of the culture plates for 24 h, and then were treated with either vehicle (DMSO) or the indicated concentrations of each one of the antiprogestins for 96 h. **(A)** Total number of cells was recorded at the beginning of the experiment and after 96 h of treatment. The growth of the treated groups was expressed as percentage of control. **(B)** Viability was assessed with the Guava ViaCount application in a similar experimental approach to that shown in the left panel when assessing cell growth. **(C)** Following a similar treatment protocol as above cells were collected, fixed in 4% paraformaldehyde, stained with propidium iodide, and analyzed by cytometry using the Guava cell cycle application. Bars, mean ± s.e.m. (n = 3).

Figure 4 shows that the six antiprogestins studied inhibited the growth of both IGROV-1 and IGROV-1 PTES cells in dose-related manners. The magnitude of the growth inhibition ranged with IC50s from ~11 to 35 μM depending upon the compounds (Figure 4A, and Table 2). The growth inhibition effect did not change for each compound in between the sibling cells, except for a slight, yet significant decline in the ORG-31710 and CDB-4124 IC50s for the PTES cells when compared to the parental cells (Table 2). When we studied the lethality of the antiprogestins towards IGROV-1 and IGROV-1 PTES we observed the six compounds impaired the viability of the cells when used at concentrations equal to or higher than 15 μM. The antiprogestins with higher lethality, as indicated by their manifestation at lower concentrations, were RU-38486, ORG-31710 and CDB-2914, when compared to the CDB-4124 derivatives that displayed lethality only at concentrations over 30 μM (Figure 4B and C). Overall, the effect of the antiprogestins was similar in IGROV-1 and IGROV-1 PTES, suggesting that their anti-cancer effect is independent of the intrinsic sensitivity to CDDP and PTX displayed by the ovarian cancer cells.

**Table 2:** Antiprogestin-induced growth inhibition toward CDDP and PTX resistant cells

**Cells**	**RU-38486**	**ORG-31710**	**CDB-2914**	**CDB-4124**	**OH-4124**	**CDB-4453**
IGROV-1	11.7 ± 1.80	21.6 ± 1.10	15.5 ± 1.10	35.5 ± 3.90	28.6 ± 3.40	30.6 ± 2.70
IGROV-1 PTES	10.7 ± 1.20	15.2 ± 1.40^c^	12.5 ± 1.10	21.3 ± 1.80^c^	25.9 ± 2.40	25.6 ± 2.60
SKOV-3	15.7 ± 1.90	17.2 ± 4.20	31.5 ± 3.10	43.6 ± 5.10	84.2 ± 9.80	44.9 ± 4.60
SKOV-3 PTES	16.0 ± 2.10	19.2 ± 2.70	31.2 ± 1.80	47.4 ± 3.90	58.4 ± 8.30	52.9 ± 2.10

Data shown are the IC50 (μM) expressed as the mean ± s.e.m (n = 3); OH-4124 indicates 17-α-hydroxy CDB-4124. ^c^p < 0.05 (Student’s *t*-test) compared to parental cells.

**Figure 5:**
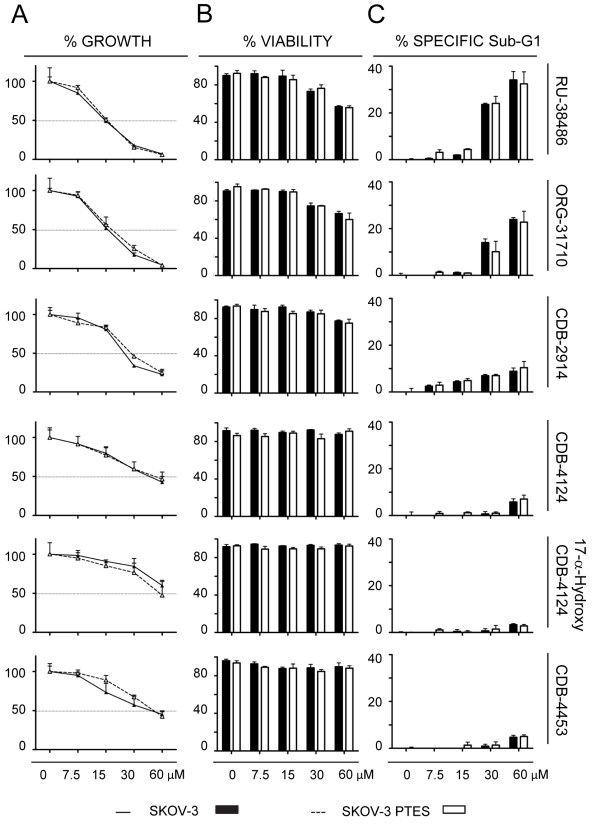
**Growth inhibition and lethality of antiprogestins towards SKOV-3 and SKOV-3 PTES cells.** Experiments were similar to those described in Figure 4, but with a different pair of cell lines. Percent cell growth **(A)**, percent viability **(B)** and percent specific Sub-G1 **(C)** were assessed for both cell lines in response to six different antiprogestins. Bars, mean ± s.e.m. (n = 3).

In Figure 5 we show the response to antiprogestins of the ovarian cancer cells SKOV-3 and their derivatives SKOV-3 PTES less sensitive to CDDP and PTX. Similarly to what it was found with IGROV-1 cells, both SKOV-3 and SKOV-3 PTES were impaired in their growth and viability by antiprogestins in a dose-related manner, without displaying major differences in the responses among them (Figure 5A). The magnitude in the IC50s for antiprogestins in the SKOV-3 cell line pair displayed a larger range when compared to the IGROV-1 pair, expanding from ~15 μM to 84 μM (Table 2). The most potent compounds in terms of lethality were RU-38486 and ORG-31710, with CDB-2914 and CDB-4124 derivatives having lesser effects (Figure 5B and C).

**Figure 6:**
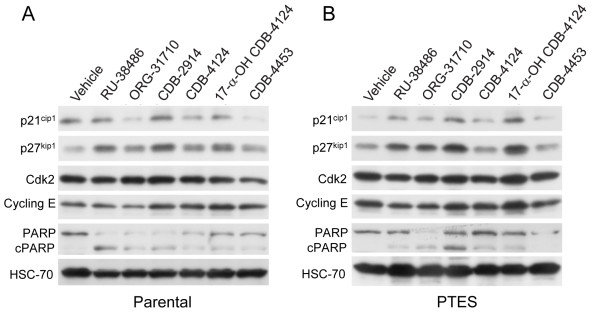
**Effect of antiprogestins on the expression of p21**^**cip1**^**, p27**^**kip1**^**, Cdk2, cyclin E, and PARP in IGROV-1 (A) and IGROV-1 PTES (B) cells.** Cells were treated with 30 μM antiprogestins for 48 h, whole cell extracts were isolated, electrophoresed, transferred to a PVDF membrane, and exposed to the indicated antibodies. HSC-70, a highly conserved protein that belongs to the HSP70 family of molecular chaperones, was used as control for protein loading.

To further study the effect of antiprogestins on cytostasis and lethality towards ovarian cancer cells of similar genetic backgrounds but different, double sensitivity to CDDP and PTX, we cultured IGROV-1 and IGROV-1 PTES in the presence of a fixed dose of 30 μM antiprogestins, which for these cells represents the limiting concentration between the induction of cytostasis and lethality depending on the compound used (Figure 4C). Results in Figure 6 show that exposure to the said concentration of the compounds for 48 h caused an increase in the abundance of the cell cycle inhibitor p27^kip1^, which was more notable for RU-38486, ORG-31710, CDB-2914 and 17-α-hydroxy CDB-4124 in both parental and PTES cells. The cyclin dependent kinase inhibitor p21^cip1^ also increased in response to the antiprogestins, yet this increase was more marked in PTES cells versus parental cells when comparing treatment versus vehicle. The increases in p21^cip1^ and p27^kip1^ by antiprogestins are consistent with cell cycle arrest associated with growth inhibition. No major differences were observed in the expression of G1 regulatory proteins Cdk2 and cyclin E in response to antiprogestins between parental and PTES cells. In terms of signs of lethality, the cleavage of PARP was observed in both IGROV-1 and IGROV-1 PTES cells in response to the compounds that had more potency in terms of reducing cellular viability (Figure 4C), with RU-38486, ORG-31710 and CDB-2914 displaying greater cleavage of PARP when compared to the CDB-4124 derivatives (Figure 6).

## Discussion

Many studies with chemoresistant ovarian cancer cells in vitro have been done using cells obtained from patient’s ascites and that are not chemotherapy naïve. For instance, PEO4 cells were obtained from a patient that received platinum-based therapy nine months earlier and display a ~8 fold resistance to CDDP when compared to their platinum sensitive counterparts PEO1 cells [16,17]. Another example is the SKOV-3 cell line, which is considered semi-resistant to platinum in vivo as was obtained from a patient that did not respond to the maximal tolerated dose of platinum [18]. The chemoresistance of these cells developed within the in vivo environment of the patient, can be considered clinically relevant and is usually reported to involve between 2- to 5-fold increases in their IC50 values when compared to the parental cells (reviewed in [19]). However, there are also various cell line pairs that were made resistant to platinum-therapy by stepwise exposure to CDDP in vitro, and, because they are highly stable, such as the OV2008 and OV2008/C13 or the A2780 and A2780/CP70 siblings, they have been very useful to study mechanisms of chemoresistance in the laboratory [20]. Yet, because these cells exhibit over 8-fold increase in resistance, they should be considered less relevant from a clinical standpoint [12,19]. Furthermore, there is significant heterogeneity in the type of resistance patients develop after front line platinum-taxane chemotherapy, including patients that show high sensitivity, others that show sensitivity to one agent but resistance to the other, or patients that show resistance to both drugs [21].

We decided to develop cell lines with resistance to both platinum and taxanes, within the range of clinical relevance. We utilized the IGROV-1 and SKOV-3 cell lines as IGROV-1 cells were previously reported to be capable of acquiring cross-resistance to PTX when made resistant to CDDP [22], whereas SKOV-3 cells, with clinically relevant endogenous resistance to CDDP, were made PTX resistant upon pulse selection [23]. We generated clinically relevant IGROV-1 and SKOV-3 cells resistant to CDDP and PTX by exposing them to both drugs in six pulse-selection challenges. We termed the IGROV-1 and SKOV-3 derivatives IGROV-1 Platinum Taxane EScape or PTES and SKOV-3 PTES, respectively, which showed double resistances in the range of 2-7 folds compared to their parental cells. We then asked if the sibling cell lines depict cross-resistance to the anti-growth effect of antiprogestins. Indeed we confirmed that all antiprogestins utilized in the study (i.e., mifepristone, ORG-31710, CDB-2914, CDB-4124, 17-α-hydroxy CDB-4124 and CDB-4453) blocked the growth of the parental and resistant derivatives cells with overall similar potency, with cytostasis manifested at concentrations lower than 15 μM, and lethality manifested at doses higher than 15 μM. These results are in agreement with a previous study we performed using the antiprogestin mifepristone in OV2008 cells and compared its effect against that observed in the highly resistant OV2008/C13 siblings. Mifepristone killed cells when used at doses higher than 20 μM, whereas at lower doses, it caused cytostasis that was reversed when the drug was removed from the culture [8]. The anti-growth effect of mifepristone was also independent of the presence of the tumor suppressor p53, because it did not discriminate between A2780 wt 53 platinum sensitive cells and A2780/CP70 platinum resistant cells carrying a mutant version of p53 [24-26], IGROV-1 cells with p53 wt expression [27], or SKOV-3 reported to carry a single nucleotide deletion in the p53 gene leading to no expression of p53 [28-31].

Mifepristone is one of the most popular antiprogestins ever developed. It was synthesized in the early 1980’s as an antiglucocorticoid but soon afterward was found to block the transcriptional activity of progesterone receptors (PR). Because of such an antiglucocorticoid effect, new generation antiprogestins were developed aiming at reducing antiglucocorticoid activity while maintaining or enhancing antiprogesterone activity. Such compounds include ORG-31710 and the CDB family members studied here, CDB-2914 and CDB-4124. The differences between the compounds are in the substitutions localized at the positions 11β and 17α. Whereas the dimethylaminophenyl substitution at the 11β-position seems to confer antiprogestin activity [32-34], modifications in position C-17 are mostly geared at modifying the antiglucocorticoid receptor activity of the compounds. Thus, ORG-31710 and the CDB derivatives are considered to have potent antiprogestin activity with less antiglucocorticoid activity when compared to mifepristone [35,36]. Of these compounds, mifepristone has been approved in the US to terminate early pregnancy (working as an antiprogestin) or ameliorating the hyperglycemia in patients with endogenous Cushing’s (working as an antiglucocorticoid) (reviewed in [37]). CDB-2914 (ulipristal) and CDB-4124 (proellex) are currently under intense investigation to assess their capacity to mitigate signs and symptoms associated with increased cell growth in endometriosis and uterine fibroids (reviewed in [38]).

We have found that mifepristone, the compound with the highest antiglucocorticoid effect, is the most potent against the growth of ovarian cancer cells either sensitive or resistant to the combination CDDP/PTX. The new generation antiprogestins represented by the CDB compounds are effective, but with a higher IC50. It is interesting to note that either the 17-α-hydroxylated or demethylated forms of CDB-4124 did not show superior potency over CDB-4124 in terms of growth inhibition, suggesting that the anti-growth effect of the molecules resides in a yet to be identified functional group.

CDB-4124 and the putative mono-demethylated metabolite CDB-4453 are all potent antiprogestins but have limited antiglucocorticoid activity when compared against mifepristone or CDB-2914 [35,39]. Because the anti-growth potency of the CDB derivatives was slightly reduced when compared to that of mifepristone or ORG-31710, these results suggest that the antiprogestin function of the molecule may be unrelated to its anti-growth capacity. Indeed we have shown that cancer cells of different tissues of origin and different degrees of hormone-dependency, such as MCF-7 breast cancer cells carrying PR, MDA-MB-231 breast cancer cells with no PR expression, PR negative and androgen receptor positive LNCaP prostate cancer cells, and PR negative androgen receptor positive PC3 prostate cancer cells are all inhibited by mifepristone with similar potency [40], strongly suggesting that the presence of the PR is not required for the inhibition of cancer growth triggered by antiprogestins. Further supporting this hypothesis it was shown that mifepristone blocked the growth of estrogen receptor negative and PR negative MDA-MB-231 breast cancer cells [41].

In another line of reasoning, it is possible that the antiglucocorticoid effect of the molecules may have some role in the antigrowth effect, as all cell lines being studied express glucocorticoid receptors (GR) [40]. The human *NR3C1* gene undergoes alternative splicing generating two main isoforms, GRα and GRβ. Considerable evidence indicates that the GRα isoform drives GR-mediated transactivation activity, whereas GRβ is a natural dominant negative inhibitor of GRα; however, GRβ can directly regulate genes not controlled by GRα (reviewed in [42]). We have shown that mifepristone blocked the growth of cancer cells with very low expression of GRα, such as OVCAR-3 ovarian cancer cells, MCF-7 breast cancer cells, and U-2OS osteosarcoma cells [40], suggesting that the presence of GRα may not be required for the antigrowth effect of antiprogestins. It remains to be determined, however, whether GRβ, which was reported capable of binding mifepristone [43], plays a role in the anti-growth effect of antiprogestins, as this receptor isoform seems to be present in all cell lines we studied so far [40].

## Conclusions

Antiprogestins of different generations with higher or lesser antiglucocorticoid activity can block the growth of ovarian cancer cells that have been made resistant to CDDP and PTX in a clinically relevant manner. Although the molecular mechanisms driving the growth inhibition by antiprogestins requires more detail, it is clear that the drugs could be developed further for anti-ovarian cancer therapy, in particular for those cases that show upfront resistance to standard of care, or for recurrent patients with platinum and/or taxane resistant disease. Due to the low toxicity of these drugs, their potential use as maintenance therapy or antirepopulation therapy following standard of care is anticipated. In this regard we have demonstrated that mifepristone was capable of abrogating the regrowth of cancer cells that escaped CDDP [9] or the combination CDDP/PTX therapy [10]. The results presented herein highlight the fact that other antiprogestins in addition to mifepristone can be efficacious against platinum/taxane double-resistant ovarian cancer cells. Whether or not ovarian cancer cells may develop resistance to antiprogestin therapy after exposure to the drugs for long periods of time, remains to be investigated.

## Abbreviations

CDDP: Cisplatin; PTX: Paclitaxel; DMSO: Dimethyl sulfoxide; CDP: Combination dose proportion; FRA: Fold resistance acquired; PTES: Platinum taxane escape cells; PR: Progesterone receptors; GR: Glucocorticoid receptors.

## Competing interests

The authors declare that they have no competing interests.

## Authors’ contributions

CGL and CMT conceived and designed the experiments. AAG performed initial validation experiments with CDB-4124 derivatives. CGL developed the drug-resistant cell lines and performed a comprehensive study on their responses to a panel of antiprogestins. MBH contributed with the western blot assays. CMT contributed with the reagents, materials and analysis tools. CGL and CMT wrote the paper. All authors approved the final version of the manuscript.
